# Synthesis of hydroxyapatite/polyethylene glycol 6000 composites by novel dissolution/precipitation method: optimization of the adsorption process using a factorial design: DFT and molecular dynamic

**DOI:** 10.1186/s13065-023-01061-7

**Published:** 2023-11-08

**Authors:** K. Azzaoui, S. Jodeh, E. Mejdoubi, B. Hammouti, M. Taleb, G. Ennabety, A. Berisha, M. Aaddouz, M. H. Youssouf, S. Shityakov, R. Sabbahi, M. Algarra

**Affiliations:** 1 Laboratory of Engineering, Electrochemistry, Modeling and Environment, Faculty of Sciences, Sidi Mohamed Ben Abdellah University, 30000 Fez, Morocco; 2 Department of Chemistry, An-Najah National University, Nablus, Palestine; 3 Laboratory of Applied Chemistry and Environment, Faculty of Sciences, Mohammed First University, 60000 Oujda, Morocco; 4 Euro-Mediterranean University of Fes, BP 15, 30070 Fes, Morocco; 5 Department of Chemistry, Faculty of Natural and Mathematics Science, University of Prishtina, 10000 Prishtina, Kosovo; 6 Department of Bioinformatics, Würzburg University, 97074 Würzburg, Germany; 7 Laboratory of Development and Valorization of Resources in Desert Zones, Higher School of Technology, Ibn Zohr University, Laayoune, Morocco; 8 INAMAT2 - Institute for Advanced Materials and Mathematics. Department of Science, Public University of Navarre, Campus de Arrosadia, 31006 Pamplona, Spain

**Keywords:** Hydroxyapatite, Adsorption, Factorial design, Molecular dynamic, Pollution, Precipitation

## Abstract

In this work, we presented a synthesis of a composite based on HAp and PEG 6000 using a new method of synthesis dissolution precipitation to be applied for application of wastewater purification from toxic metal ions. Multiple characterization methods were used to analyze the morphology and the structure of the well-prepared compounds including FT-IR, Raman, XRD, XPS, TGA and SEM were used to conduct a composite analysis. The adsorption effectiveness of this analysis towards Pb^2+^ and various other hazardous metal ions found in sewage was assessed. Batch experiments were conducted to optimize the various operational parameters including adsorbent dose, temperature, pH, contact time, and initial concentration. The Langmuir isotherm was used to fit the data, and it predicted monolayer adsorption with a maximum capacity of 67 mg g^−1^ for HAP PEG600 and 60 mg g^−1^ for HAp. A pseudo-second-order equation fits the adsorption process well (0.961–0.971). The thermodynamic data support the spontaneous metal bonding to the composite receptor sites. Theoretical calculations showed that the interaction strength is very strong and gets stronger when the PEG6000 is deprotonated. The results presented here are supported by evidence acquired from experiments. Theoretical computation using Monte Carlo (MC) and Molecular Dynamic (MD) simulation models showed excellent affinity of prepared foams for the model ion Pb^2+^ with highly negative adsorption energy values indicating vigorous interactions of Pb^2+^ with the adsorbate surfaces.

**Supplementary Information:**

The online version contains supplementary material available at 10.1186/s13065-023-01061-7.

## Introduction

Hydroxyapatite (HAp), which has the chemical formula Ca10(PO4)6(OH)2,. HAps have structural properties which give them great adaptability in terms of their suitability for substitution both on the calcium site and on the phosphorus or hydroxyl site. HAp is commonly used as a food additive, a nutritional supplement, and in the manufacture of fertilizers, animal feed, and dental and bone implants [1–3].

HAp is the inorganic mineral phase present in vertebrate bones and teeth. Synthetic HAp is found in two different structures, hexagonal and monoclinic, depending on the preparation conditions [4]. Posner et al. (1957) first proposed the space group of hexagonal HAp as P63/m with the lattice parameters of a = 9.4224 A°, b = a, c = 6.8825 A°, and λ = 119.97°. Mirror planes at z = ¼, z = ¾, and a sixfold axis of rotation give rise to the P63/m space group (m is indication of the mirror planes perpendicular to the c-axis). The sixfold axis is perpendicular to the (001) faces of HAp and parallel to the c-axis. This sixfold Polyethylene Glycol (PEG) is a polymer condensation of ethylene oxide and water. PEG is used in emulsifying agents, detergents, soaps, plasticizers, ointments, and other products. Whereas the chemical and physical characteristics of PEG are well understood [5]. Yet, because of their practical applications, it is vital to investigate a few physical parameters such as ultrasonic velocity, viscosity and thus adiabatic compressibility, free length, and so on. Rotation axis is a screw axis, meaning that within the unit cell, half way along the c-axis, the pattern is repeated after each 60° rotation [6].

**Fig. 1:**
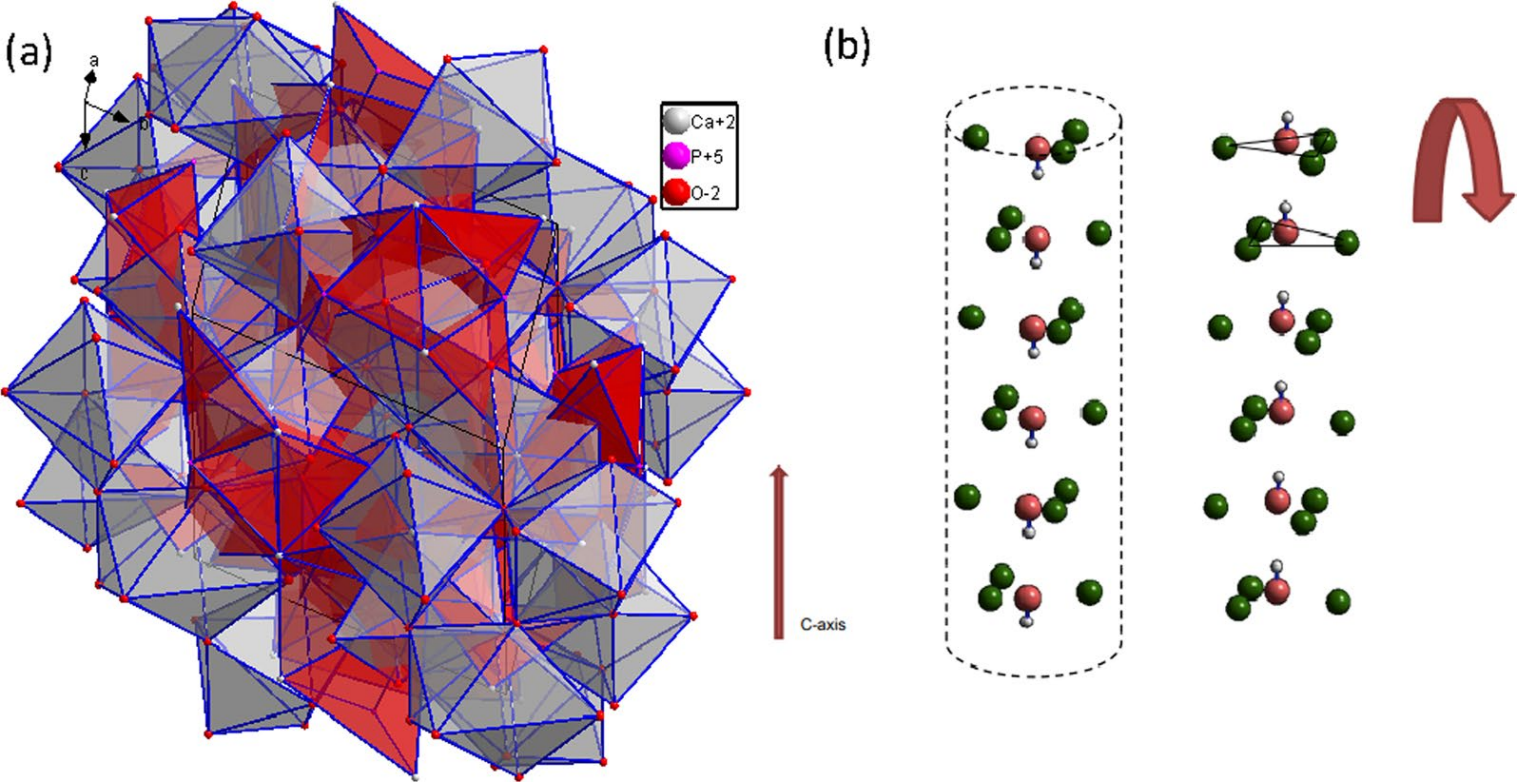
Hydroxyapatite structure. Hydroxyapatite planar view of (001) face (**a**). There are two positions available for calcium ions as labeled CaI and CaII. Hydroxyapatite calcium channel (**b**). The CaII cations at the position z = ¼ and ¾ form triangles, these triangles are rotated in each successive mirror plane by 60°, giving rise to calcium channels that are occupied by hydroxyl groups in HAp. Calcium is in green and hydroxyl in pink

**Fig. 2:**
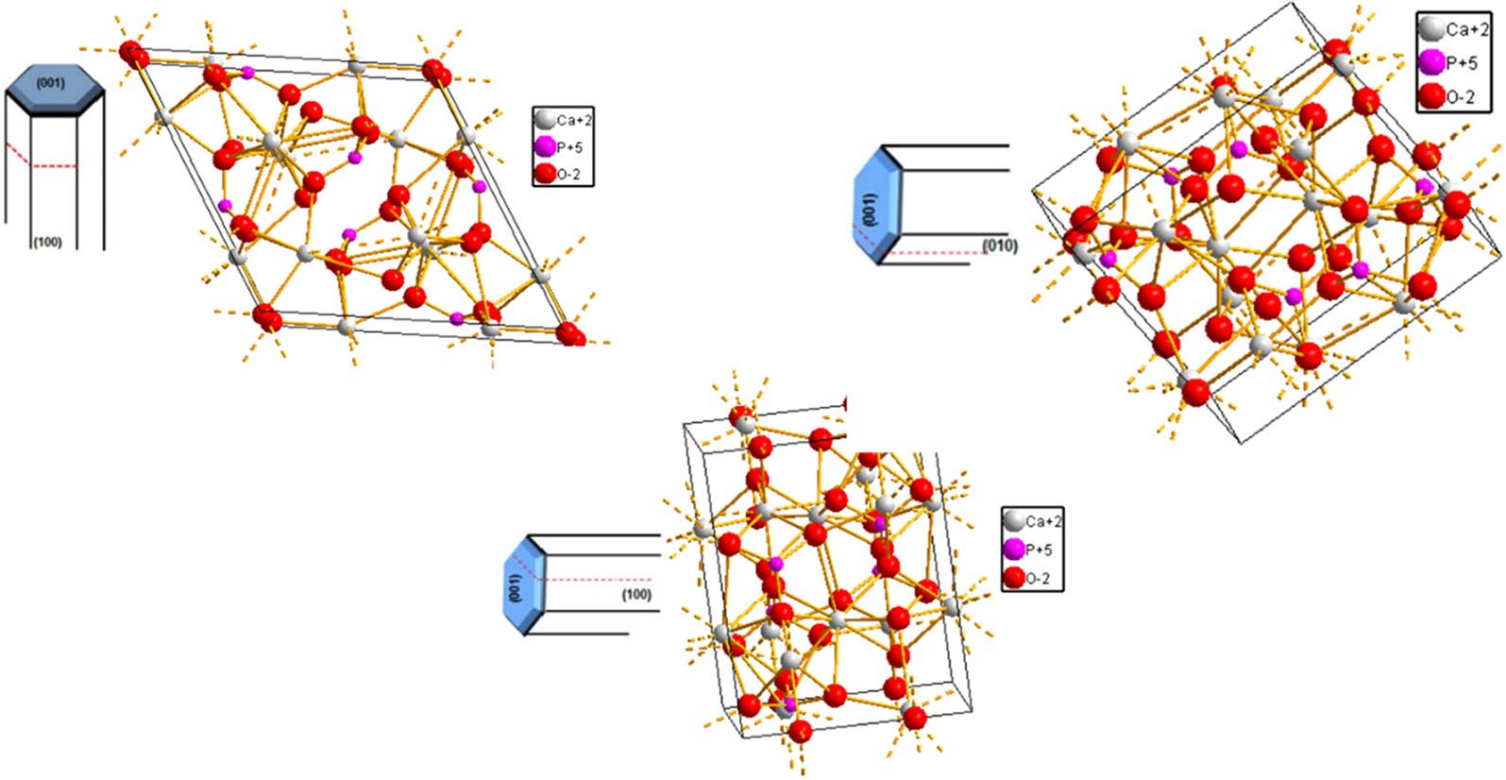
Hydroxyapatite different crystallographic orientations chemistry

The general formula of HAp is Ca_10_(PO_4_)_6_(OH)_2_ with OH having the possibility to be substituted by Cl or F. There are two distinct positions in HAp for the Ca ions (Fig. 1a). Ca(I) cations are in 4f (1/3,2/3,Z) position and coordinated with 9 oxygen atoms from 6 phosphate groups to form tricapped trigonal prisms [7]. Ca(II) cations are in 6 h special position and coordinated with 7 oxygen atoms. 6 of the coordinated oxygen atoms to the Ca(II) cations are from 5 different phosphates tetrahedral and one is from a hydroxyl group. The Ca(II) cations, at positions z = ¼ and ¾, form triangles, which are rotated in each successive mirror plane by 60° and give rise to what is known as calcium channel (Fig. 1b). The calcium channels are occupied by hydroxyl groups in HAp (Fig. 2) [8]. Hydroxyl groups are reflected in the mirror planes (HO|OH), in each column, along the c-axis, and are disordered in hexagonal structure. Later, Elliott et al. (1973) found that there is another structure available for HAp other than the hexagonal structure known as monoclinic HAp. The monoclinic HAp is proposed to have a space group of P21/b with lattice parameters of a = b = 9.42 A°, c = 6.88 A°, and λ = 120°.

The crystal structure of apatite shown above is particularly tolerant of distortions and is known to be able to accommodate more than half of the Table 1 [9]. Thus, many cations, both divalent (Sr, Pb, Ba, Mn, Mg, Fe, Zn, Ni, Cd.), monovalent (Na, K, Li), trivalent (rare earths, Y) or even tetravalent (Th, U) can substitute for Ca^2+^ ions, with variable affinities for each of the two crystallographic sites Ca1 and Ca2 [10]. The phosphate tetrahedral can be replaced by other anionic groups such as AsO_4_^3−^, SO_4_^2−^, CO_3_^2−^, and SiO_4_^4−^. we can replace OH anion with another substitution like F^−^ and Cl^−^.

**Table 1:** Space group, position of the X anion and lattice parameters of the three constituent minerals of the apatite family [11]

	Space group	Anion position X	Mesh Parameters (Å)	
			a,b	c
Fluorapatite	P6_3_/m	0,0,1/4	9.397	6.878
Hydroxyapatite	P2_1_/b	0,0,0.1979	9.417	6.875
Chlorapatite	P2_1_/b	0,0,0.4323	9.598	6.776

In this work, we used a new method of co-precipitation for the synthesis of a HAp-PEG 6000 composite for possible application as material for Environmental. In this work, the use of materials, such as HAp-PEG 6000, as adsorbents for environmental remediation has received great attention. This composite represents unused resources and presents serious disposal problems. We demonstrated that such materials are capable of selectively adsorbing metal ions. Then we tried to work on the theoretical part using Monte Carlo (MC) and Molecular Dynamic (MD) simulation models showed excellent affinity of prepared foams for the model ion Pb2 + with highly negative adsorption energy values indicating vigorous interactions of Pb2 + with the adsorbate surfaces.

## Materials and methods

### Chemicals

Hydroxyapatite (HAp) was synthesized according to a reported procedure [8]. Calcium nitrate tetrahydrate Ca(NO_4_)_2_, 4H_2_O, ammonium dihydrogen phosphate NH_4_HPO_4_, ammonium hydroxide (NH_4_OH), and Polyethylene glycol (MW 6000), were purchased from Sigma-Aldrich, were all analytical grade with purity more than 99%, Distilled water.

### Synthesis of HAp-PEG_6000_ composite

**Fig. 3:**
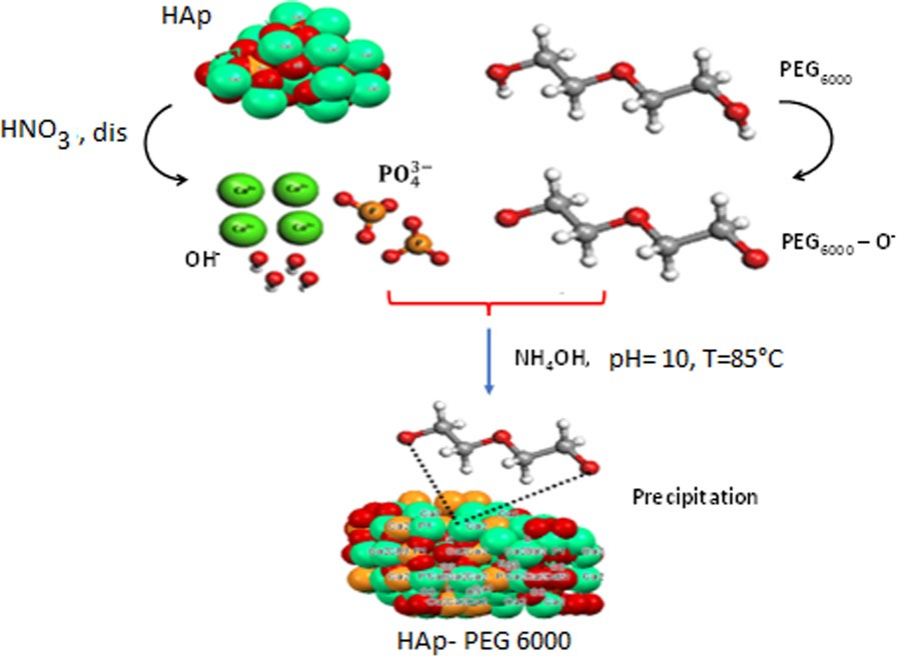
Schematic illustration of the HAp and HAp-PEG_6000_ preparation

The synthesis of porous HAp was made by the wet chemical process, according to a procedure already reported [12]. In brief, two solutions were prepared individually. Solution A contained 14.82 g Ca(NO_4_)_2_, 4H_2_O in 200 mL of distilled water, and Solution B had 13.80 g NH_4_HPO_4_ in 100 mL of distilled water. The solutions were mixed well for 90 min at RT until everything was dissolved. After 48 h of mixing, the combined solutions were vacuum filtered and dried for a night at 100 °C. The synthesis of Hap-PEG_6000_ composite takes two phases. During the experiments, 5 g of stoichiometric hydroxyapatite was put into deionized water and then treated with 2 M nitric acid at pH 3 to dissolve it. This process resulted in a translucent solution called S1, which indicated that the structure of HAp had been broken down. Afterward, 0.5 g of PEG 6000 was dissolved in 30 ml of deionized water and added dropwise into the S1 solution, which was then mixed for 30 min. The pH was promptly elevated to 10.5 in the second step using ammonium hydroxide (NH_4_OH), resulting in weight precipitation (Fig. 3). The product was obtained in a closed system at temperature 85 °C to prevent the emission of CO_2_ into the atmosphere. The process for creating HAp-PEG 6000 using a dissolution–precipitation technique can be seen in Fig. 3. The products we obtained were calcined at 900 °C for 2 h to obtain a porous hydroxyapatite-based final product.

### Characterization

FTIR spectra were acquired using a Shimadzu FTIR-8400S analyzer to identify the functional groups in HAp and Hap-PEG6000 composites. The FTIR spectra were acquired using the KBr pellet method with a resolution of 2 cm^−1^ in the 4000 to 400 cm^−1^ range. The crystallinity and phase compositions of the precipitated and Hap-PEG6000 composites were determined using a Shimadzu XRD-6000 X-ray diffractometer with CuKa1 radiation and a 0.02 step size. The Scherrer equation was used to calculate the crystallite size of HAp and HAp-PEG6000composite. To determine the crystallite length and average width/thickness, two XRD diffraction peaks (002) and (310) were utilized. The Quanta 200 FEG Scanning Electron Microscope was utilized to examine the morphology and chemical content of the powders produced. The XRD analysis of the HAp and Hap-PEG_6000_ composite created in this study displayed peaks that were characteristic of the hydroxyapatite structure (JCPDS 9–432). Using CuKa1 radiation in the scanning range of 2θ = 10–70° with a step size of 0.02 and a Shimadzu XRD-6000 X-ray diffractometer, the phase compositions and crystallinity of the precipitated and HAp/PEG 6000 composite were determined. The crystallite size of HAp and HAp/PEG 6000 composite was calculated.

Raman spectroscopy is based on the interaction of light with the chemical bonds of a substance. This yields detailed information about chemical structure, polymorphism, crystallinity and molecular dynamics. Renishaw inVia spectrometer was used to collect Raman spectra with the range of 300–1800 cm^−1^, recording 5 times for 10 s of each accumulation, a wavelength of 532 nm, backscattering configuration using a microscope with a 100  × objective, 100% power of green laser line of argon, and acquisition time of 10 s. The samples were subjected on Al foil in order to remove the fluorescence background which is a challenge in the Raman analysis of HA.

The prepared materials were characterized with TGA-DTA using the *Shimadzu DTG-60 & Shimadzu TA-*60 by heating the furnace components and argon cleaning gas. The TGA-DTA test aims to see the process that occurs, which is characterized by a reduction in the weight of the composite during the heating time and temperature of 25–900 °C.

Chemical analysis was performed by means of X-ray photoelectron spectroscopy (XPS). Spectra were acquired in ultra-high vacuum (5.0 × 10^–9^ mbar) with an XR50 Mg anode source operating at 150 W and a Phoibos 150 MCD-9 detector (D8 advance, SPECS Surface Nano Analysis GmbH, Germany). Spectra were recorded at pass energy of 25 eV with a step size of 1.0 eV for survey spectra and 0.1 eV for high resolution spectra of C1s, O1s, Si2p, Cl2p, N1s, and Ti2p. C1s peak was used as a reference. CasaXPS software (Casa Software Ldt, UK) was used for the determination of atomic elemental composition applying the manufacturer set of relative sensitivity factors. Two samples per condition were analyzed.

### DFT calculation

In the course of this study, all DFT calculations were carried out utilizing DMol3 in conjunction with the M06-2X functional [13, 14]. By numerically applying atomic basis sets in terms of the double numerical plus polarization function (DNP) basis set version 3.5, a global orbital cutoff of 4.4 was used [15, 16]. Limit of 1 × 10^–5^ Hartree were used for the geometry optimization’s convergent tolerances of the energy and displacement (Ha). Klamt’s Conductor-like Screening Model (COSMO) is utilized to account for the solvent, which in this case is water [17, 18], to investigate the interaction of the Pb^+2^ ions with PEG 6000 (in neutral and deprotonated form). The adsorption energy (E_ads_) was calculated by the following equation [19–23]:

1 $${E}_{adsorption}={E}_{Pb(II){}_{||}PEG }-\left({E}_{Pb(II)}+{E}_{PEG}\right)$$

where ${E}_{Pb(II){}_{||}PEG}$ is the total energy of the interacting entities, E_Pb(II),_ and *E*_*PEG*_ is the total energy of the entities.

### Adsorption

As a representative metal ion, Pb^2+^ was selected for this study. The batch method was used for the adsorption procedure. Adsorption was carried out at 25 °C with varied volumes of foam and metal ion solutions ranging in concentration from 40 to 50 mg/L. The effects of adsorption time and pH were investigated, and the pH was altered by the addition of HNO_3_ or NaOH. The thermodynamic parameters were used to determine the nature of the adsorption process. Metal ion content changes were determined using AAS flame atomic absorption spectroscopy. The adsorption capacity of composites was calculated using Eqs. (2) and (3) [4].

2 $$\mathrm{\%Removal}=\frac{{C}_{0}-{C}_{e}}{{C}_{0}}\  \cdot  100$$

3 $${Q}_{e}=\frac{{C}_{0}-{C}_{e}}{W}V$$

where C_0_ denotes the beginning metal ion and C_e_ denotes the equilibrium metal ion concentration in ppm. Q_e_ is the equilibrium adsorption capacity in ppm, W is the absorbent foam weight in mg, and V is the solution volume in L.

#### Isotherm of the adsorption process

The Langmuir and Freundlich isotherm models were followed in this study to conclude the adsorption behavior of Pb^2+^ ions on HAp and HAp/PEG 6000 composite [4]. The Langmuir isotherm model is presented in Eqs. (4) and (5) [36–38]:

4 $$\frac{{C}_{e}}{{Q}_{e}}=\frac{1}{{q}_{max}}{C}_{e}+\frac{1}{{q}_{max}{K}_{L}}$$

where C_e_ is the Pb^+2^ concentration in ppm, Q_e_ is the amount of metal ion removed per unit mass of HAp-PEG6000 or HAp at equilibrium (mg/g), q_max_ is the foam's greatest single layer adsorption capacity (mg/g), and K_L_ (L/mg) is the Langmuir constant.

The Langmuir isotherm model can be used to predict whether adsorption will be favorable or unfavorable by utilizing the dimensionless constant separation factor given in Eq. (4) [36–38].

5 $${\mathrm{R}}_{\mathrm{L }}=\frac{1}{1+ {K}_{L }{C}_{e}}$$

where C_0_ represents the initial Pb^+2^ and K_L_ represents the Langmuir constants. If the R_L_ value exceeds one, the adsorption is considered unfavorable; otherwise, it is considered favorable or linear if it is between one and one.

The heterogeneous surface energy of non-ideal adsorption process is represented by the Freundlich isotherm model indicated in Eqs. 6 and 7 [36–38].

6 $$\mathrm{ln}({q}_{e })=\mathrm{ln}{k}_{f}+\frac{1}{n}\mathrm{ln}{C}_{e}$$

7 $$ {\text{Q}}_{{\text{e}}} = {\text{ K}}_{{\text{F}}} {\text{C}}_{{\text{e}}}^{{{1}/{\text{n}}}} $$

where 1/n represents the adsorption intensity and K_F_ denotes the relative adsorption capacity [12]. Adsorption is advantageous if 1/n is between 0.1 and 0.5; it is unfavorable if 1/n.

#### Kinetics of the adsorption process

The pseudo first-order and second-order kinetic models, described below, were used to study the rates of Pb^2+^ adsorption on the surfaces of HAp-PEG 6000 and HAp. Equations 8, 9, 10, 11 were employed to compute the linearized versions of the rate equations [4] [36–38].

8 $$\mathrm{ln}({q}_{e }- {q}_{t})=\mathrm{ln}{q}_{e}-\mathrm{K}1\mathrm{ t}$$

9 $$\frac{\mathrm{t}}{{\mathrm{q}}_{\mathrm{t}}}=\frac{1}{{\mathrm{K}}_{2}{q}_{e}^{2}}+\frac{\mathrm{t}}{{\mathrm{q}}_{\mathrm{e}}}$$

10 $$ {\text{Q}}_{{\text{t}}} = {\text{ K}}_{{{\text{id}}}} {\text{t}}^{{{1}/{2}}} + {\text{ Z}} $$

11 $${ln}\frac{K({T}_{2 })}{K({T}_{1})}=\frac{Ea}{R} \cdot   (\frac{1}{{T}_{1}} - \frac{1}{{T}_{2}})$$

12 $$ {\text{ln}}\left( {{1} - {\text{F}}} \right) \, = \, - {\text{K}}_{{{\text{fd}}}} *{\text{t}} $$

where Q_t_ denotes temperature-dependent adsorption capacity and q_e_ denotes equilibrium adsorption capacity (mg/g).

K_1_ represents the pseudo-first-order rate constant (min), and K_2_ represents the pseudo-second-order rate constant (g/mg. min). Z (mg/g) can be used to calculate the boundary layer thickness, where Kid is the diffusion rate constant measured in mg/g.min^1/2^.

In Eq. (12), k_fd_ (min) represents the rate of film diffusion, and F represents the fractional accomplishment of equilibrium (F = q_t_/q_e_).

The entropy (S°), enthalpy (H°), and Gibbs free energy (G°) were calculated using Eqs. 13, 14, 15 [36–38].

13 $$ {\text{K}}_{{\text{c}}} = {\text{ C}}_{{{\text{ads}}}} /{\text{C}}_{{\text{e}}} $$

14 $$ \Delta {\text{G}}^{0} = \, - {\text{RTlnK}}_{{\text{c}}} $$

15 $$\mathrm{ln}{K}_{s}= \frac{\mathrm{\Delta S}^\circ }{R}-\frac{\mathrm{\Delta H}^\circ }{RT}$$

where T represents the solution temperature (K), R represents the ideal gas constant (J/mol K), C_ads_ represents the equilibrium Pb^+2^ adsorbed quantities (mg/L), C_e_ represents the equilibrium concentration (mg/L).

## Result and discussion

### XRD analysis

**Fig. 4:**
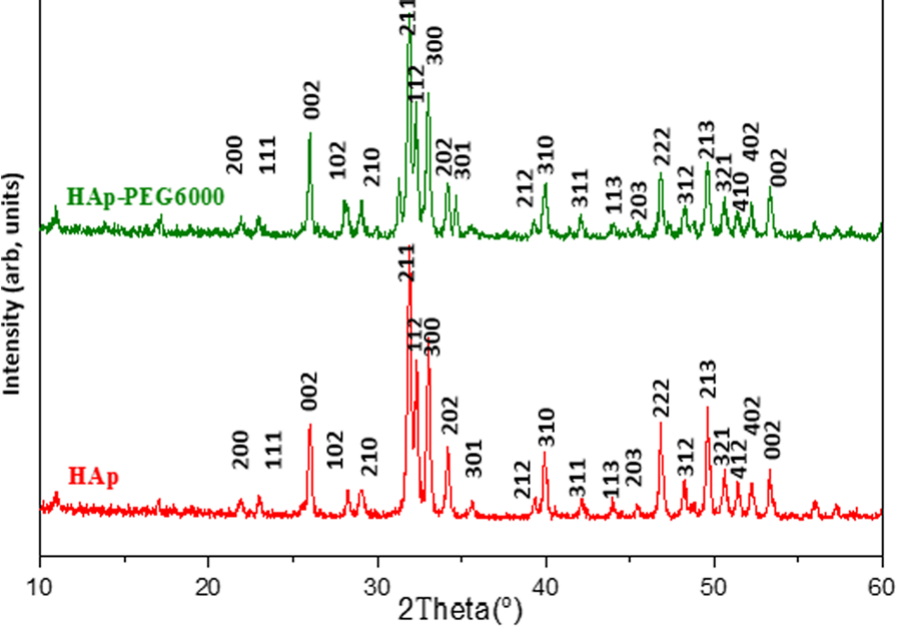
X-ray diffraction of HAp and HAP-PEG 6000 composite

The X-ray diffraction patterns of hydroxyapatite and hydroxyapatite modified with polyethylene glycol are displayed in Fig. 4. The patterns reveal the presence of well-crystallized hydroxyapatite. After heat treatment at 900 °C for 2 h, the X-ray patterns of the powders display a single phase of HAP, which is consistent with the hydroxyapatite ASTM data (JCPDS) file (no. 09–0432). There are no indications of impurities such as calcium hydroxide or calcium phosphates, which suggests that phase-pure HAp was synthesized under the experimental conditions. The diffraction peaks in the planes (0 0 2), (2 1 1), (1 1 2), and (3 0 0) are strong and narrow, indicating that the HAp crystallizes effectively.

### FTIR analysis

**Fig. 5:**
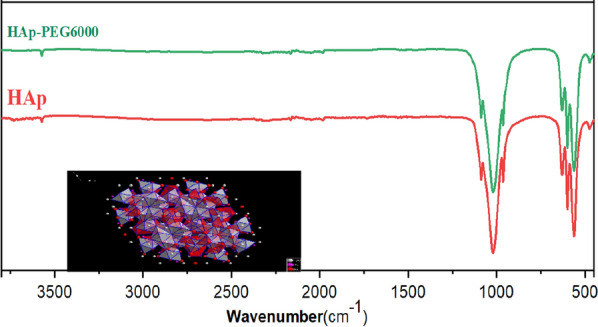
FTIR spectra of HAp and HAP-PEG 6000 composite

The FTIR spectra of HAp and HAp modified with PEG 6000 are displayed in Fig. 5. The small bands observed at 3572 and 632 cm^−1^ correspond to the vibration of the hydroxyl (O–H) group, while those observed at 1089, 1045, and 962 cm^−1^ correspond to the stretching vibration of phosphate, and the bands at 601 and 570 cm^−1^ are due to the vibration of phosphate. Based on FTIR analysis, the presence of PEG 6000 has no impact on the structural deformation of HAp.

When PEG 6000 is dissolved in an aqueous solution, a PEG-OH bond is formed. This bond has the ability to chelate Ca^2+^ and attract it from Ca(NO_3_)_2_, 4H_2_O to form the bond of PEG–O–Ca^2+^–O– PEG. Next, PEG–O–Ca^2+^–O–PEG reacts with the PO_4_^3−^ released from (NH_4_)_2_HPO_4_ to produce the HAp crystal nucleus. In this process, the release rates of Ca^2+^ and PO_4_^3−^ are crucial factors. The experiments indicate that when PEG 6000 is absent or at a low concentration, many deposits are formed quickly, indicating rapid HAp production. As the concentration of PEG increases, the initial deposits decrease, and it takes longer to produce a large quantity of deposits, indicating that PEG 6000 reduces the release rate of Ca^2+^ and restrains the formation of HAp crystal nucleus. When the release rate of Ca^2+^ and the deposit rate of HAp crystal nucleus reach a dynamic equilibrium, the HAp crystal nucleus deposits isotopically, and the spherical HAp particles are ultimately obtained. However, when the concentration of PEG 6000 is low, the release rate of Ca^2+^ is very fast, and many HAp crystal nucleuses are produced, which cannot deposit isotopically in the precipitation center, resulting in the as-prepared HAp particles having a non-spherical morphology.

### Scanning electron microscopy (SEM)

**Fig. 6:**
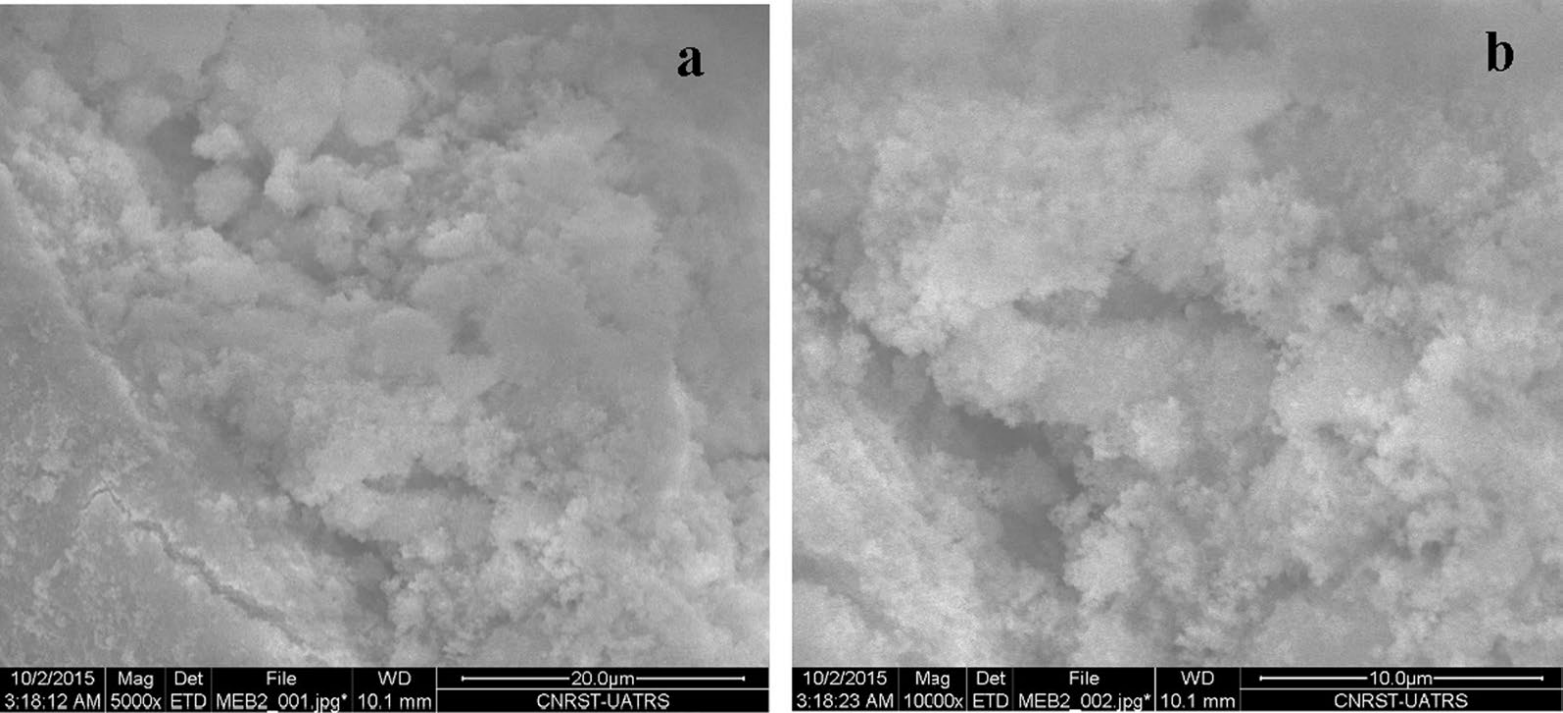
SEM micrographs of HAP-PEG 6000 at 900 °C (**a**) and SEM micrographs of pure HAp at 900 °C (**b**)

In Fig. 6, SEM micrographs of HAp treated with PEG show small agglomerated particles with an average diameter of 50–60 nm. The pores in the image are favorable for the circulation of physiological fluid in bone implantation. Figure 6a illustrates the morphology of clusters of grain structure for pure HAp sintered at 900 °C for 2 h in stagnant air. Figure 6b shows the powder modifier by PEG 6000 obtained after a 2 h heat treatment at 900 °C of the HAp of agglomerated grains.

### Raman spectra

**Fig.7:**
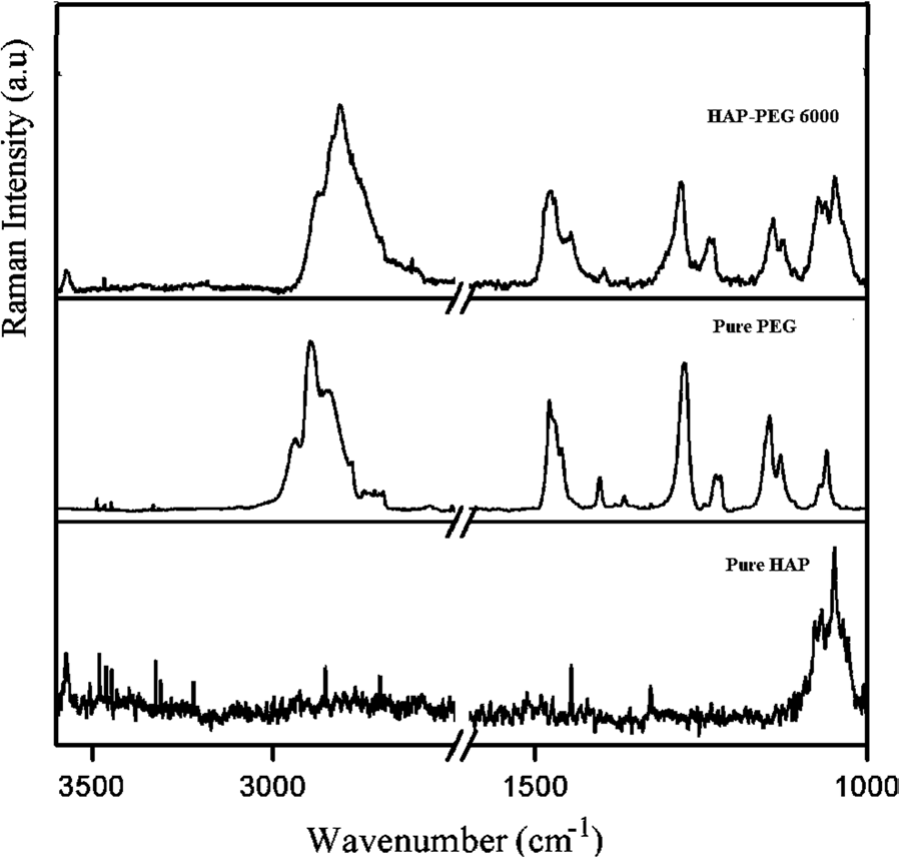
Raman spectra of pure HAp, pure PEG and HAp-PEG 6000 in the region 3600–1000 cm^-1^

Surface functionalization is the introduction of chemical functional groups on the surface of HAp. Non-covalent and covalent grafting techniques are available for functionalizing the surface of HAp with PEG. The non-covalent technique uses electrical characteristics for nonspecific PEG surface adsorption on HAp. The covalent procedure involves grafting by direct chemisorption. The Raman spectra of pure HAp, pure PEG, and HAp-PEG 6000 are shown in Fig. 7. The Raman spectra of all samples reveal peaks of both PEG and HAp, confirming the combination of PEG with HAp. The Raman active vibrational modes of HAp show no significant shift, indicating that the structure of HAp remains unaffected by the addition of PEG 6000. This suggests that the crystallinity of HAp nanoparticles is not lost during the functionalization process. When a hydrogen bond is created in a molecule, the stretching vibration in the Raman spectrum shifts red and the bending vibration shifts blue.

In PEG functionalized HAp, the symmetric stretching vibrations of the methylene group of PEG (2895 and 2845 cm^−1^) are red-shifted by around 12 and 6 cm^−1^, respectively. In all functionalized samples, a new band at 2905 cm^−1^ is observed. In surface-modified samples, this is due to the creation of hydrogen-bound antisymmetric stretching vibrations of the methylene group of PEG. The skeleton vibrations of PEG exhibit a red shift of roughly 4 cm^−1^ in the Raman spectra of PEG functionalized HAp. When compared to the pure PEG Raman spectra, the C-O stretching vibrations exhibit a red shift in all samples. In the PEG functionalized samples, there is also a blue shift for the CH_2_ twisting vibrations.

### HAp-PEG composite TGA-DTA analysis

**Fig. 8:**
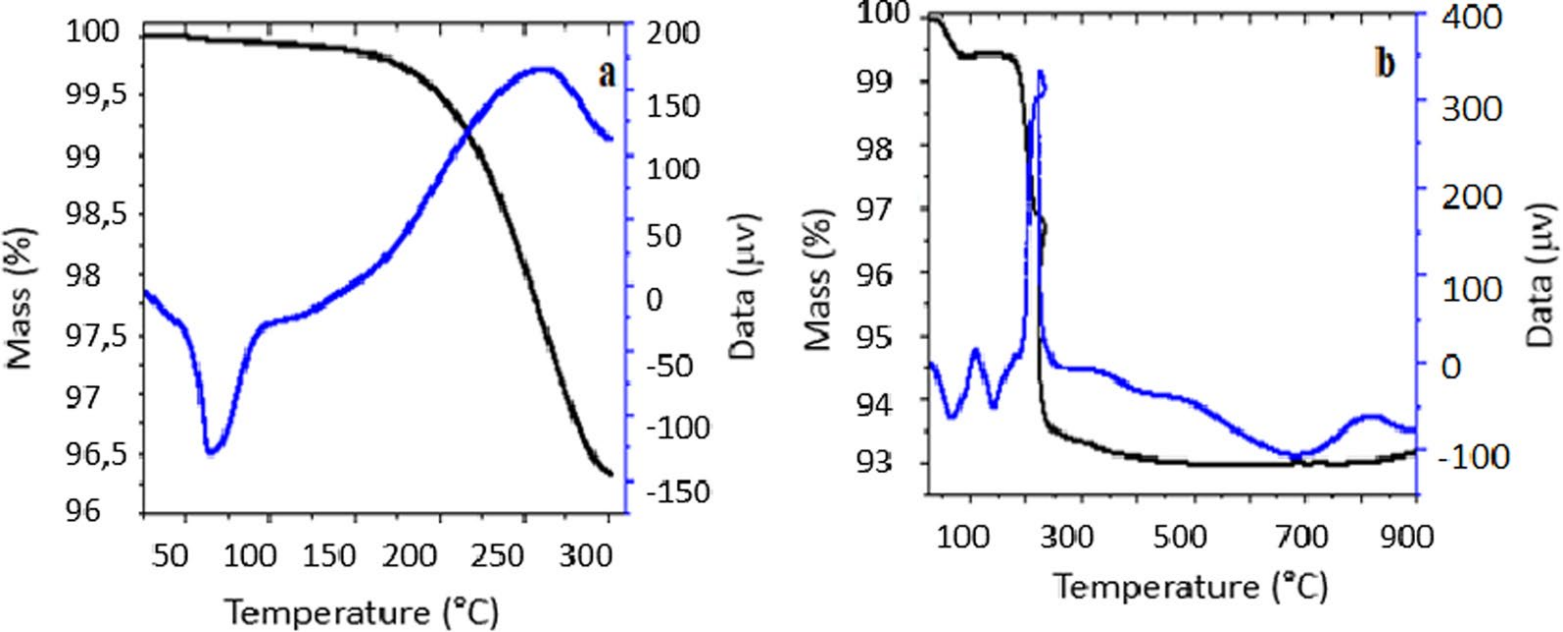
TGA-DTA analysis pure HAp (**a**) and HAP-PEG 6000 composite at 60 ℃ (**b**)

The TGA and DTA techniques were used to determine the weight loss of the HAp-PEG 6000 composite sample, which was carried out within a temperature range of 25–900 °C. Figure 8 illustrates the TGA-DTA plot of the HAp-PEG 6000 composite sample synthesized at 60 °C, where the pure HAp sample shows gradual weight loss. The weight loss of the pure HAp sample was measured at a starting temperature of 25 °C and a starting mass of 10 g. The first weight loss occurred between 52.5 and 172 °C, which resulted in a mass decrease of 0.65% due to the elimination of water molecules, as confirmed by DTA. Then, at 170.5–300 °C, there was a significant weight loss of up to 3.57%, connected with the dehydration reaction of the C–OH group, resulting in an overall weight loss of 4.21% in pure PEG, leaving a residue of 6.42 g. The weight loss of the HAp-PEG 6000 composite was observed at 75 °C, and the composites were tested at a temperature of 25 °C and a weight of 10 g. The first weight loss in the composite sample occurred as much as 0.52% at a temperature of 25–110 °C, which was connected with the elimination of water molecules adsorbed on the sample's surface. The second weight loss, validated by DTA, occurred at a temperature of 178–305 °C and was associated with the dehydration reaction of the C–OH group. The third weight loss occurred at temperatures ranging from 330 to 485 °C, as much as 0.31%, related to the breakdown of HAp, emitting CO_2_ gas. The total weight reduction in TGA was 6.96%, with a residual mass of 3.94 g in the composite. When compared to pure HAP, the decomposition temperature of the HAp-PEG 6000 composite fell gradually in weight [21, 22].

### X-ray photoelectron spectroscopy analysis

**Fig. 9:**
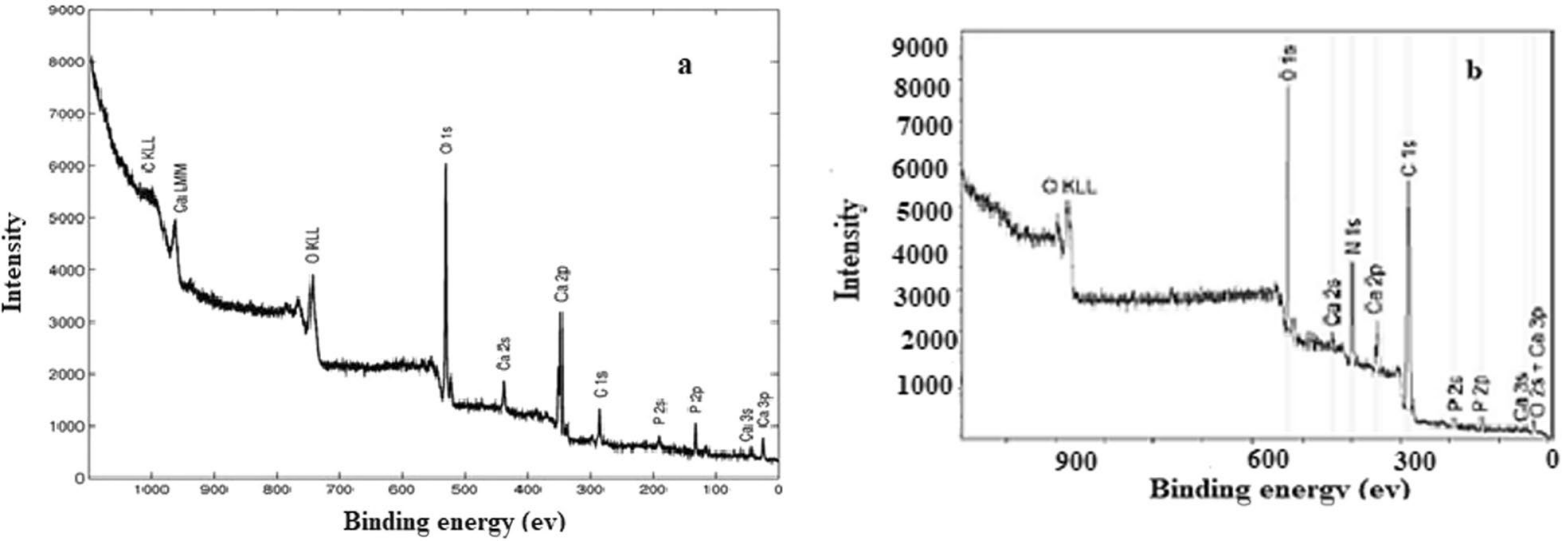
XPS spectra of Hap (**a**), and HAp-PEG 6000 (**b**)

Figure 9 presents the spectra of hydroxyapatite and hydroxyapatite modified by PEG. The figure indicates that those elements like O, P and Ca are detected in the sample of hydroxyapatite.

Additional elements also found like N, C, and O which assure the existence of some coupling agents and polymer. The data of spectra of found elements are summarized in Table 2. The presence of four different types of carbon bonds is confirmed by carbon atom (C 1 s). The bonds of C–C and C–H are represented by the peak at binding energy of 285 eV and this usually happened at the surface of the modified hydroxyapatite. The other bonds of ether groups C–O–C and carbonyl C–O are shown at peaks of binding energy of 286.20 eV and 288.23 eV. Some impurities present during synthesis due to the presence of CO_2_ in air. The other signal of Ca 2p signal represents two lines: Ca 2p 1/2 due to calcium apatite and Ca 2p 3/2 belongs to a simple Ca-O bond. The presence of C-N bond was confirmed from the signal that occurs at 398.83 eV. Both phosphate oxide and OH groups are assigned at the binding energy of 530.90 eV and 533.5 eV, while PO_4_^−3^ group results in the presence of the spectrum of the P 2p bond at 134.08 eV.

**Table 2:** Elemental compositions, binding energy, peak area and bonds of HAp-PEG 6000 by XPS analyses

Name	Binding energy/eV	Area/a.u	Content/at. %	Bond
HAp-PEG 6000				
C 1 s	283.70	2651.18	65.98	C–C, C–H
C 1 s	285.20	853.32	22.94	C–O–C
C 1 s	286.47	383.74	10.43	C=O
C 1 s	287.80	189.50	4.64	–CO3
O 1 s	532.90	3463.16	87.67	PO_4_^3−^, Ca–O
O 1 s	534.48	532.13	14.33	P–OH
Ca 2p 3/2	347.30	572.03	101.00	Ca–O
Ca 2p 1/2	351.85	286.01	–	
N 1 s	399.85	1322.772	101.00	N–C
P 2p	135.08	165.60	101.00	PO_4_^3−^

### Surface characterization using N_2_ adsorption technique.

The surface morphology of HAp and HAp-PEG 6000 studied by the BET method, before and after and after changes and given in Table 3. The surface area of HAp decreased after PEG-grafting from 32 to 23 m^2^ g^*−*1^ and the the pore size slightly decreased from 0.25 cm^3^g^−1^ to 0.18 nm cm^3^g^−1^. Looking at the main results of the present study, it can be stated that HAp was successfully precipitated in the presence of PEG 6000 using novel dissolution/ precipitation method. In fact, through this synthesis process we demonstrated that apatite interacts with. Furthermore, we observed that PEG 6000 can controls the crystallinity and crystal growth of HAp as well as its surface specific, pore size and pore volume.

**Table 3:** Data obtained by nitrogen adsorption study

	BET surface area m^2^g^−1^	Pore size cm^3^g^−1^
HAp	32	0.25
HAp-PEG 6000	23	0.18

Prior to BET surface area measurement samples are usually degassed at specified temperature for desired duration. Choice of temperature might be related to thermal stability of material under investigation, which on the other hand can be guessed by measuring corresponding TGA analysis. However, in certain cases some materials does not have enough thermal stability, at slightly higher temperature or prolong heating at low temperature they may experience phase transformation.

The test was started with 150–200 ℃ for 3 h. Then the temperature was increased from 200 to 250 ℃ and used the same time. Then we prolong degassing time to 4 h for both temperatures.

### Adsorption mechanism

Previous research has shown that hydroxyapatite-based compounds can adsorb metal ions, such as Pb^2+^, through ion exchange, with elimination of high concentrations of ions occurring via a dissolution–precipitation mechanism rather than diffusion. Hydroxyapatite has been found to be effective in removing lead from polluted solutions [24], and while some researchers have suggested that the sorption and removal of lead using hydroxyapatite is limited to the surface, others have found that the process using HAp-PEG 6000 may involve multiple mechanisms [5, 21].

The ion exchange mechanism occurs when metal ions from the polluted solution replace the Pb^+2^ ions in the solid phase of hydroxyapatite. This process involves the dissolution of apatite followed by precipitation as shown in ng Eq. 16:

16 $$ {\text{Ca}}_{{{1}0}} \left( {{\text{PO}}_{{4}} } \right)_{{6}} \left( {{\text{OH}}} \right)_{{2}} - {\text{C}}_{{{\text{2n}}}} {\text{H}}_{{{\text{4n}} + {2}}} {\text{O}}_{{{\text{n}} + {1}}} + {\text{ x M}}^{{{2} + }} \,\,\,\,\, \to \,\,\,\,{\text{Ca}}_{{{1}0 - {\text{x}}}} {\text{M}}_{{\text{x}}} \left( {{\text{PO}}_{{4}} } \right)_{{6}} \left( {{\text{OH}}} \right)_{{2}} - {\text{C}}_{{{\text{2n}}}} {\text{H}}_{{{\text{4n}} + {2}}} {\text{O}}_{{{\text{n}} + {1}}} + {\text{ x Ca}}^{{{2} + }} $$

In addition to ion exchange, metal ions can also be adsorbed or attached to existing cationic gaps on the surface of HAp-PEG 6000. Another mechanism involves complexation of metal ions on the surface of HAp, followed by dissolution–precipitation. The removal of metal ions in this case involves two steps: dissolution of HAp-PEG 6000, followed by precipitation of a metal phosphate as shown in Eqs. 17–18:

17 $$ {\text{Ca}}_{{{1}0}} \left( {{\text{PO}}_{{4}} } \right)_{{6}} \left( {{\text{OH}}} \right)_{{2}} + {\text{ 12 H}}^{ + } \,\,\,\, \to \,\,\,\,{1}0{\text{ Ca}}^{{{2} + }} + {\text{ 6H}}_{{2}} {\text{PO}}^{{{4} - }} + {\text{ 2OH}}^{ - } + {\text{ C}}_{{{\text{2n}}}} {\text{H}}_{{{\text{4n}} + {2}}} {\text{O}}_{{{\text{n}} + {1}}} \left( {{\text{dissolution}}} \right) $$

18 $$ {1}0{\text{ M}}^{{{2} + }} + {\text{ 6H}}_{{2}} {\text{PO}}^{{{4} - }} + {\text{ 2OH}}^{ - } + {\text{ C}}_{{{\text{2n}}}} {\text{H}}_{{{\text{4n}}}} {\text{O}}_{{{\text{n}} + {1}}} \,\, \to \,\,{\text{M}}_{{{1}0}} \left( {{\text{PO4}}} \right)_{{6}} \left( {{\text{OH}}} \right)_{{2}} - {\text{C}}_{{{\text{2n}}}} {\text{H}}_{{{\text{4n}} + {2}}} {\text{O}}_{{{\text{n}} + {1}}} + {\text{ 12 H}}^{ + } \left( {{\text{precipitation}}} \right) $$

The hyperbolics HAp of the data-derived Langmuir isotherm plot give asymptotically to a constant limit value. Based on the Langmuir classification, the shape of the curve indicates that it is a type I isotherm. This suggests that the adsorbate is only adsorbed onto a single layer of the matrices. Once the first layer is formed, the solute–solvent interactions become stronger than the solute-surface interactions.

The HAp-PEG 6000 based composites were designed to contain many sites with high affinity for metal ions (Additional file 1: Fig. S1a). The metal binding sites include hydroxyl groups [25]. Additionally, the 3D HAp-PEG 6000 model integrating the full molecular size of PEG (n $\approx$ 100) was built to observe the PEG nanoparticle interaction with the HAp slab (Additional file 1: Fig. S1b, S1c). The molecules were covalently linked to each other via the ether bond as Ca^2+^–O–PEG. Before the covalent attachment, the interaction energy of − 624.12 kcal/mol was established between the analyzed substances. Moreover, the predicted possible binding site (heavy metals, ligands, etc.) was defined on the top of the HAP-PEG 6000 molecule interacting with the Pb nanoparticle (fcc, 4.92) with the London free energy of − 1.97 kcal/mol. This binding site can be also implemented for remediation to adsorb heavy metal ions, such as Cd^+2^, Zn^+2^, Pb^+2^, and Cu^+2^ (Additional file 1: Fig. S1c). Additionally, the Pb nanoparticle (Additional file 1: Fig. S2a) interaction with HAp-PEG 6000 was represented as electron density, contact and electrostatic maps (Additional file 1: Fig. S2c, d), where electrostatically positive regions are, by default colored blue, negative regions, red and neutral regions, white.

The HAp- PEG 6000 based composite was designed to contain many sites with high affinity for metal ions **(**Additional file 1: Fig. S2). The metal binding sites oxygen and hydroxyl groups. Pb nanoparticle (a) Additional file 1: Fig. S2a and its interaction with HAp-PEG 6000 visualized via electron density, contact and electrostatic maps are shown in Additional file 1: Fig. S2b. The Pb nanoparticle is depicted in the vdW spheres. The hydrogen atoms are omitted for all the maps. Electrostatically positive regions are, by default colored blue, negative regions, red and neutral regions, white. Additional file 1: Fig. S2c, d.

### Adsorption of Pb^2+^

The efficiency of the newly synthesized composite for removing heavy metals was tested using a batch adsorption method. The objective was to identify the ideal conditions for heavy metal adsorption. To conduct the experiments, Pb^+2^ was chosen as the representative ion.

#### Effect of pH

The pH parameter is very important since the change in pH the receptor sites can be activated to bind with adsorbate or can be blocked.

In our study 10 mL solution of adsorbate that contain 25 mg foam was mixed and stirred for 30 min.

Additional file 1: Fig S3a shows the effect of pH study**.** During a low pH (at about 2.5) all hydroxyl groups are presented in their protonated forms which reduces their affinity for adsorption of heavy metal and the adsorption efficiency decreases significantly. On the other hand, when the pH increased, all the hydroxyl groups (OH) will begin shifting from their Lewis acid forms to their Lewis base forms, and this cause shift of weak chelating agents to strong chelating agents due to the lone pair of electrons on oxygen atom. This lead to have the best adsorption efficacy occurred at a pH of 7.5. After increasing pH more than 8 the adsorption efficacy starts going down and this can be related to the generation of metal oxide which is soluble in aqueous solutions.

#### Effect of adsorbent dose

In Additional file 1: Fig. S3b, a diagram shows how the removal percentage of the adsorbent foam is affected by its dosage. The foam was used at different concentrations of Pb^+2^ ranging from 5.0 to 50.0 mg, while keeping other variables constant at a solution volume of 10.0 mL, an initial Pb^+2^ concentration of 15.0 ppm, and a pH of 4.3. The mixtures were stirred for 30 min at room temperature. The results indicate that an increase in foam dosage leads to a higher amount of metal removed. The study found that at a dosage of 25 mg, the maximum reduction of Pb^+2^ was approximately 95.0 and 75% for HAp and HAp-PEG 6000, respectively.

#### Effect of metal ion concentration

The study investigated how the concentration of lead ions affects the rate of removal while keeping other parameters constant such as solution volume, pH, time, temperature, and foam dose at 10 mL, 4.3, 30 min, 25 °C, and 15 mg, respectively. Additional file 1: Fig. S3c illustrates that the maximum removal rates for the HAp and PEG 6000 composites were 70% and 90%, respectively. However, both composites exhibited a decrease in adsorption efficacy at initial concentrations higher than 20 ppm. The results indicated that at concentrations of 20.0 ppm or lower, there were metal receptors available, and the adsorption process was governed by ion diffusion mechanism. On the other hand, at higher concentrations, the availability of metal receptors decreased due to saturation, resulting in a decrease in the adsorption efficiency [7].

#### Temperature effect on adsorption

The research investigated the effect of temperature on the percentage elimination of Pb^+2^ in a range from 15 to 45 °C. The maximum elimination was observed at room temperature, as depicted in Additional file 1: Fig. S3d. However, the efficacy of HAp and HAp-PEG 6000 reduced as the temperature increased to 45 °C, indicating that the adsorption process is exothermic and does not require heat. Although the efficacy of HAp and HAp-PEG 6000 reduced slightly, the efficacy of other foams remained stable as the temperature increased. This could be related to the presence of aromatic rings, as the literature suggests that aromatic complexation may require some heat. Metal ions typically form sandwich complexes with aryl groups.

#### Effect of contact time

The research also examined the effect of contact time on the rate of Pb^+2^ elimination while keeping other parameters constant at 10 mL, 7.5, 20 ppm, 25 °C, and 15 mg. The results presented in Additional file 1: Fig S3e indicate that the percentage clearance of Pb^+2^ increased with time until it reached equilibrium after 30 min. This finding could be explained by the availability of metal receptors, which diminish with time due to adsorption, with practically all metal receptors engaged after 30 min. The research showed that HAp and HAp-PEG 6000 have a higher metal adsorption capacity than HAp and HAp-PEG 6000. This could be due to the polymer rigidity resulting from the presence of more aromatic pendant groups, as shown in Additional file 1: Fig S3e.

### Adsorption analysis

#### Isotherm

The Langmuir and Freundlich isotherm models were presented in Additional file 1: Fig S4 used to estimate the adsorption equilibrium between Pb^+2^ ion and HAp and HAp-PEG 6000 in water, as well as to evaluate metal ion dispersion on their surfaces. The choice of isotherm used in adsorption is influenced by various parameters, including the correlation coefficient [26]. Table 4 presents the determined values of all configurable parameters from Additional file 1: Fig S4. The obtained correlation coefficients using the Langmuir isothermal (Additional file 1: Fig S4a) model are lower, indicating that the adsorption of Pb^+2^ ions follows the Langmuir equation, where they are uniformly and consistently distributed across the porous surfaces of the foam. The R_L_ separation factor (Eq. 5) ranges from 0 to less than 1 for different doses of foam adsorbents (Table 4), indicating that HAp and HAp-PEG 6000 have a high affinity for the relevant metal ions. From the value of 1/n and the correlation factor R^2^, Freundlich model (Additional file 1: Fig S4b) does not represent the adsorption process.

**Table 4:** Isotherm parameters for the adsorption of Pb^+2^ ions by HAp and HAp-PEG 6000

		HAp	HAp-PEG 6000
Langmuir isotherm	Q_0 _(mg/g)	2.271	3.039
	K_L _(L/mg)	0.112	0.042
	R^2^	0.856	0.912
	R_l_	0.68	0.72
Freundlich isotherm	1/n	0.819	0.685
	K_F _(L/mg)	22.772	28.389
	R^2^	0.991	0.996

#### Adsorption kinetics of Pb^+2^ ions on HAp and HAp-PEG 6000

The adsorption of Pb^+2^ ions by HAp and HAp-PEG 6000 composites was studied using several kinetic models (Additional file 1: Fig S5) to better understand the adsorption mechanism.

The pseudo-first (Additional file 1: Fig S5a) and pseudo-second order (Additional file 1: Fig S5b) models were used to simulate metal adsorption by foam adsorbents, and the experimental results showed that the value of R^2^ for the pseudo-second order was greater than that for the pseudo-first order on the HAp and HAp-PEG 6000. The computed q_e_ values were close to the observed q_e_ values, indicating that several rate-limiting processes may be present for the adsorption process. The initial linearity of the graphs in Additional file 1: Fig S5b suggests that Pb^+2^ adsorption on HAp and HAp-PEG 6000 composite begins with an instantaneous adsorption on the external surface, resulting in a chemical complexation between Pb^+2^ and surface functional groups. The activation energy of the adsorption was calculated, and the nearly nonexistent activation energy suggested a spontaneous adsorption mechanism [12].

#### Thermodynamics study

The estimation of standard free energy, standard enthalpy, and standard entropy parameters was necessary to understand the spontaneity and type of adsorption. ΔG^0^ (J mol^−1^) was calculated using Eq. 14, and Additional file 1: Fig S6 depicts the mapping of ln Ks vs. 1/T. Table 5 lists the various thermodynamic parameters calculated using the slopes and intercepts.

**Table 5:** Parameters obtained by the kinetic models for the composite HAp and HAp-PEG 6000

Pseudo-First-Order Kinetic Model						
	HAp			HAp-PEG 6000		
	**K**_**1**_ (g/mg.min)	**Q**_**cal**_ (mg/g)	R^2^	K_1_ (g/mg.min)	Q_cal_ (mg/g)	R^2^
**Pb**^+2^	0.246	90.260	0.913	0.253	100.836	0.968

The pseudo-second-order parameters for uptaking of Pb^2+^ ions HAp and HAp-PEG 6000						
	HAp			HAp-PEG 6000		
	**K**_**2**_ (g/mg.min)	**Q**_**cal**_ (mg/g)	R^2^	K_2_ (g/mg.min)	Q_cal_ (mg/g)	R^2^
**Pb**^+2^	1.005	3.663	0.981	0.272	3.945	0.979

Intra-particle diffusion parameters of Pb^2+^ ions(Fig. S5c) bonding onto HAp and HAp-PEG 6000						
T (K)	HAp			HAp-PEG 6000		
	K_id_	Z	R^2^	K_id_	Z	R^2^
**Pb**^+2^	23.1	10.618	0.934	27.269	7.431	0.951

Thermodynamics parameters values for the adsorption of Pb^+2^ ions onto HAp and HAp-PEG 6000						
**T(K)**	HAp			HAp-PEG 6000		
	**∆G°** (KJ/mol)	**∆H°** (KJ/mol)	**∆S°** (J/K.mol)	**∆G°** (KJ/mol)	**∆H°** (KJ/mol)	**∆S°** (J/K.mol)
**298**	− 23.077	16.268	77.494	− 19.528	12.349	65.574
**313**	− 24.239			− 20.512		
**323**	− 25.015			− 21.168		
Liquid film diffusion model						
	K_df_	R^2^				
HAp	2.094	0.918				
HAp-PEG 6000	2.218	0.942				

Table 5 and (Additional file 1: Fig S6) lists the various thermodynamic parameters calculated using the slopes and intercepts. Both HAp and HAp-PEG 6000 showed negative free energies, indicating a spontaneous uptake mechanism at a wide range of temperatures [35]. The positive values of ΔS^0^ and ΔH^0^ indicate that the adsorption process increases entropy at the solid/solution interface. The adsorption mechanism involves metal ions migrating from the solution to the outer surfaces of HAp and HAp-PEG 6000 particles, followed by diffusion across the boundary layer, and subsequent adsorption at coordination sites located on the surface of composite HAp and HAp-PEG 6000 particles. To gain a more in-depth understanding of the adsorption mechanism, the liquid film model and intraparticle diffusion model (Additional file 1: Fig S5c) were used. The transport of metal ions through a liquid film surrounding the foam adsorbent is the most extended phase of the adsorption process according to the liquid film diffusion model, as shown in Eq. 12.

If the equation reveals that the plot of ln (1–F) vs t produces a straight line that passes through the origin, then the adsorption process includes diffusion via a liquid film around the HAp and HAp-PEG 6000.

The graph in Additional file 1: Fig. S7 did not have any linear lines crossing the origin and had exceptionally low R^2^ values of 0.9459 and 0.9184 for Pb^+2^, respectively.

This implies that the step influencing velocity was not ion diffusion across the liquid film surrounding the HAp and HAp-PEG 6000. These findings show that, while not the slowest stage in determining the rate,

These findings suggest that, while not the slowest step in determining the pace, the liquid film's diffusion pattern may have an effect on the adsorption of Pb^+2^, particularly at the beginning of adsorption, as shown in Table 5.

According to the subsequent results of our teams we have always sought and improved either the synthesis method or the composites which increase the adsorption efficiency [36–38].

## Theoretical results

### Monte carlo (MC) and molecular dynamic (MD)

MC simulations are used to study the interaction between Pb^+2^ ions and the modelled HAp surface with a pre-adsorbed layer of PEG 6000. The MC calculations were performed in two stages: (1) by pre-adsorbing 2 short (5 meric) chain units the AC surface onto HAp, and b) by introducing in the system Pb^+2^ ions and solvent (water) molecules (1 Pb^+2^ ion + 350 water molecules). The HAp surface model was under Periodic Boundary Conditions with a HAp (1 − 1 0) surface and the size of: 18.842 × 13.760 × 8.371 Å^3^ with an inclusion of 35 Å vacuum layer to accommodate the adsorbent molecules and water. The very often employed Universal force field was used for the simulations MC and MD calculations [16, 17, 27, 28] performed via NVT (t = 298 K) with a total simulation time of 1 ns [29–31].

For the purpose of determining the interaction strength and geometry between the HA/PEG surface and Pb^+2^, two initial case configurations were utilized (Additional file 1: Fig S8). These included the monomeric interaction between PEG6000/Pb^+2^ in neutral and deprotonated states.

As shown in Additional file 1: Fig S8, the interaction strength is very strong and gets stronger when the PEG6000 is deprotonated. This is because the distance between the two molecules gets shorter when the PEG 6000 is deprotonated, going from 2.37 to 2.14 Å.

### Onte carlo and molecular dynamic

In order to accurately calculate the different energy outputs, it is essential to determine the adsorption configuration of the adsorbate ions that results in the best possible adsorption. The interaction of the adsorbate ions with the HAP-PEG 6000 interface provides a source of information that may be used in the calculation of the adsorption energetics associated with this method (Additional file 1: Fig S9). Quantitatively speaking, this may be performed by solving Eq. (1) to obtain the adsorption energy (Eads) [23, 26, 32, 33].

This technique for measuring molecular complexity is based on generating a large number of combinations of the species (molecules, ions) used in the simulation. These combinations are generated at random. [16, 32, 34, 35]. The adsorption geometries of the adsorbate molecules are shown in Additional file 1: Fig S10. In both of these interfaces (PEG6000 in neutral or deprotonated state onto HAp) the Pb^+2^ ions are close to the interface and interact strongly with some relatively high negative adsorption energies (Additional file 1: Fig S9) [22, 34].

The results of the experiment are corroborated by the fact that the adsorbate molecules that were placed on the HAp surface had a significantly greater negative value of Eads.

## Conclusions

The composite based on HAp and PEG 6000 by a new method of synthesis dissolution precipitation, for commercial application in wastewater purification. SEM, DRX, and FT-IR techniques were used to characterize the produced composites. the XRD pad analyzes give a well crystalline structure, our products calcined at 900 °C for 2 h, as well as the SEM analysis shows the homogeneous and spherical structure. The created HAP-PEG 6000 composite was utilized to remove hazardous metal ions from wastewater. The ideal adsorption circumstances were discovered. Multiple metal ions were quantitatively removed from the sewage sample. The metal ions Pb^+2^, which were used as model ions, were shown to adhere to pseudo-second order kinetics during the kinetic research. Thermodynamic analysis produced negative Gibbs free energy values, which suggested that Pb^+2^ spontaneously coordinated to the surface of the composite. DFT demonstrates that Pb^2+^ has a strong interaction with the PEG6000 interface. Simulations using Monte Carlo and Molecular Dynamics show that there is a considerable interaction between the Pb^+2^ ions and the surface of the HAP-PEG 6000 molecule, regardless of whether the HAP-PEG 6000 molecule is protonated or not. These results are validated by experimental evidence.

### Supplementary Information

**Additional file 1: Fig S1.** Schematic diagrams showing the HAp interaction with PEG 6000 molecule using the PEG monomer (a) and polymer (b, c). The hydrogen atoms are omitted to enhance clarity. The possible heavy metal and binding sites are depicted as dotted areas. The molecular surfaces are implemented to visualize the molecular sHApes. The Pb nanoparticle is depicted in the vdW spheres. **Fig S2.** Pb nanoparticle (a) and its interaction with HAp-PEG 6000 visualized via electron density, contact and electrostatic maps. The Pb nanoparticle is depicted in the vdW spheres. The hydrogen atoms are omitted for all the maps. Electrostatically positive regions are, by default colored blue, negative regions, red and neutral regions, white. **Fig S3.** The effect of various parameters on the removal (%) of Pb+2 by of the two composite (a) pH value (b) foam dose (c) initial [Pb+2] (d) temperature and (e) time. **Fig S4.** Langmuir (a) and Freundlich (b) adsorption plots of Pb+2 ions HAp and HAp-PEG 6000 at different temperatures. **Fig S5.** Kinetic plots of Pb+2 adsorption by composite a) Pseudo first-order, b) Pseudo-second order, and c) Intraparticle diffusion model. **Fig S6.** Adsorption thermodynamics of Pb+2 ions onto HAp and HAp-PEG 6000. **Fig S7.** plot of Liquid film diffusion model for the adsorption of Pb+2 by HAp and HAp-PEG 6000. **Fig S8.** Optimized geometries, adsorption energies and r[(O) – Pb+2] distances for the interaction of Pb+2 ions and the PEG6000 in neutral and protonated state. **Fig S9.** Lowest energy geometries derived from MC and MD for the Pb+2 ions adsorbed onto interface of Hap /PEG6000 (in neutral and deprotonated form). **Fig S10.** Probability distribution of the adsorption energies from MC for the Pb+2 ions adsorbed onto interface of Hap/PEG6000 (in neutral and deprotonated form).

## Data Availability

Adequate and clear descriptions of the applied materials and tools are provided in the materials and method section of the manuscript. In addition, the obtained data is justifed by mentioning the figures and tables in the manuscript. In general, all data generated or analysed during this study are included in this published article and its Additional file.
