# Supplementary material for: Synthesis of hydroxyapatite/polyethylene glycol 6000 composites by novel dissolution/precipitation method: optimization of the adsorption process using a factorial design: DFT and molecular dynamic
Source: BMC Chem. 2023 Nov 8;17(1):150. doi: 10.1186/s13065-023-01061-7 (PMC10634111; doi:10.1186/s13065-023-01061-7)
Supplement: Supplementary file 1 — Additional file 1: Fig S1. Schematic diagrams showing the HAp interaction with PEG 6000 molecule using the PEG monomer (a) and polymer (b, c). The hydrogen atoms are omitted to enhance clarity. The possible heavy metal and binding sites are depicted as dotted areas. The molecular surfaces are implemented to visualize the molecular sHApes. The Pb nanoparticle is depicted in the vdW spheres. Fig S2. Pb nanoparticle (a) and its interaction with HAp-PEG 6000 visualized via electron density, contact and electrostatic maps. The Pb nanoparticle is depicted in the vdW spheres. The hydrogen atoms are omitted for all the maps. Electrostatically positive regions are, by default colored blue, negative regions, red and neutral regions, white. Fig S3. The effect of various parameters on the removal (%) of Pb+2 by of the two composite (a) pH value (b) foam dose (c) initial [Pb+2] (d) temperature and (e) time. Fig S4. Langmuir (a) and Freundlich (b) adsorption plots of Pb+2 ions HAp and HAp-PEG 6000 at different temperatures. Fig S5. Kinetic plots of Pb+2 adsorption by composite a) Pseudo first-order, b) Pseudo-second order, and c) Intraparticle diffusion model. Fig S6. Adsorption thermodynamics of Pb+2 ions onto HAp and HAp-PEG 6000. Fig S7. plot of Liquid film diffusion model for the adsorption of Pb+2 by HAp and HAp-PEG 6000. Fig S8. Optimized geometries, adsorption energies and r[(O) – Pb+2] distances for the interaction of Pb+2 ions and the PEG6000 in neutral and protonated state. Fig S9. Lowest energy geometries derived from MC and MD for the Pb+2 ions adsorbed onto interface of Hap /PEG6000 (in neutral and deprotonated form). Fig S10. Probability distribution of the adsorption energies from MC for the Pb+2 ions adsorbed onto interface of Hap/PEG6000 (in neutral and deprotonated form). [file 13065_2023_1061_MOESM1_ESM.docx]

| 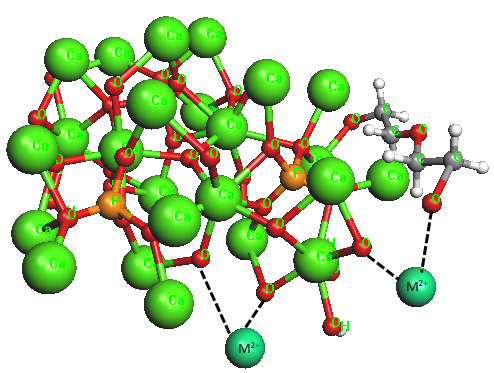  **(a)** | **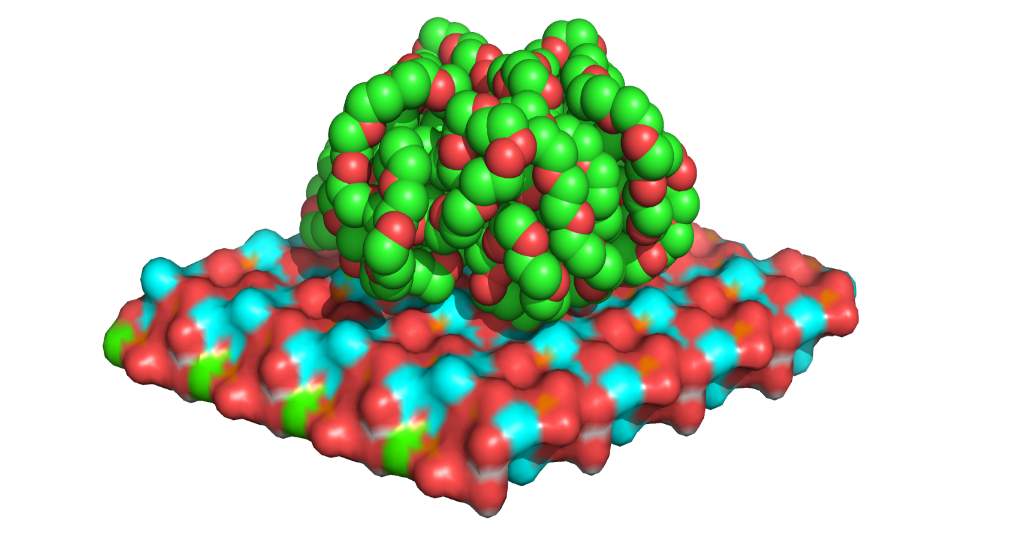**  **(b)** |
| --- | --- |
| 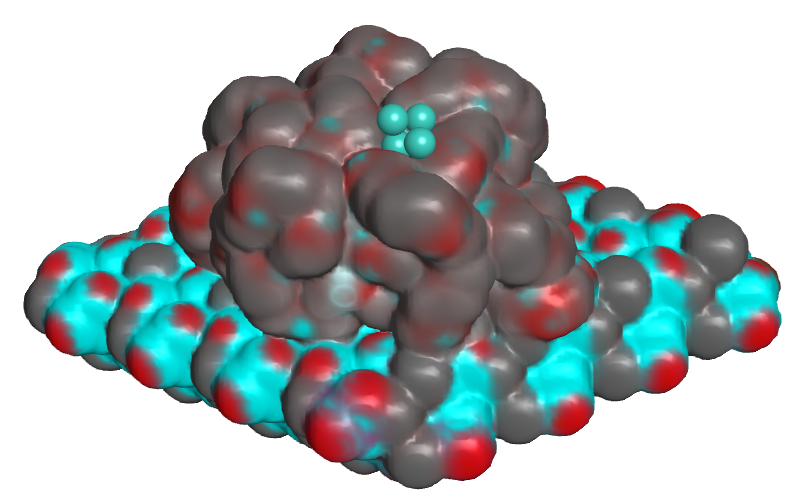  **(c)** | |

**Fig. S1.** Schematic diagrams showing the HAp interaction with PEG 6000 molecule using the PEG monomer (a) and polymer (b, c). The hydrogen atoms are omitted to enhance clarity. The possible heavy metal and binding sites are depicted as dotted areas. The molecular surfaces are implemented to visualize the molecular sHApes. The Pb nanoparticle is depicted in the vdW spheres.

| 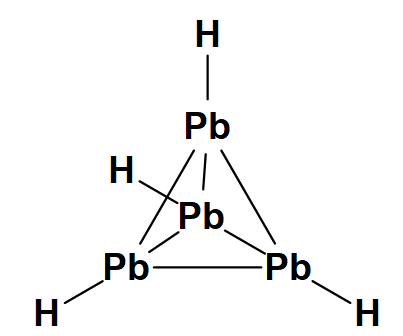  **(a)** | 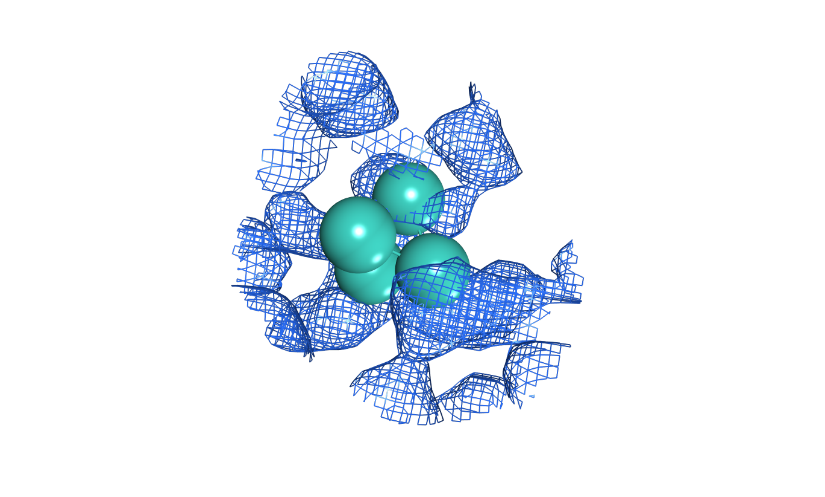  **(b)** |
| --- | --- |
| 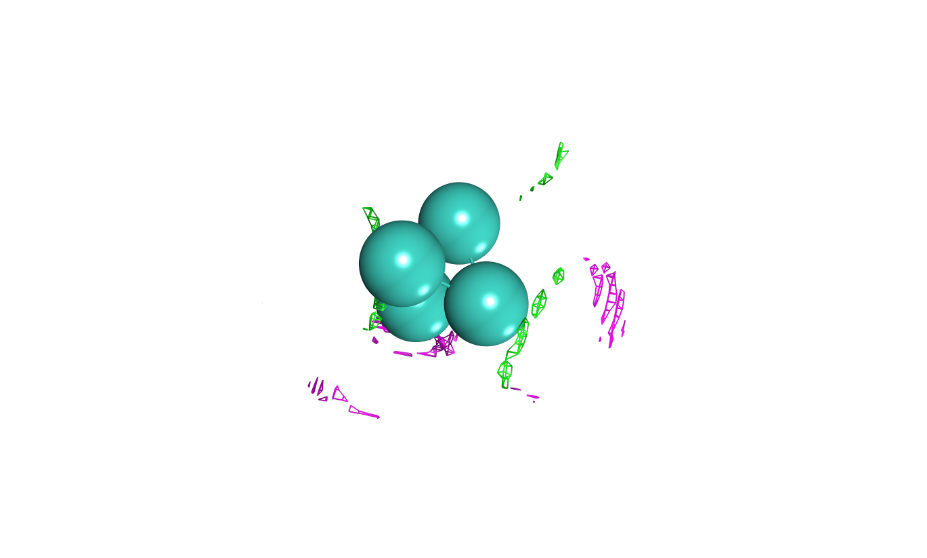  **(c)** | 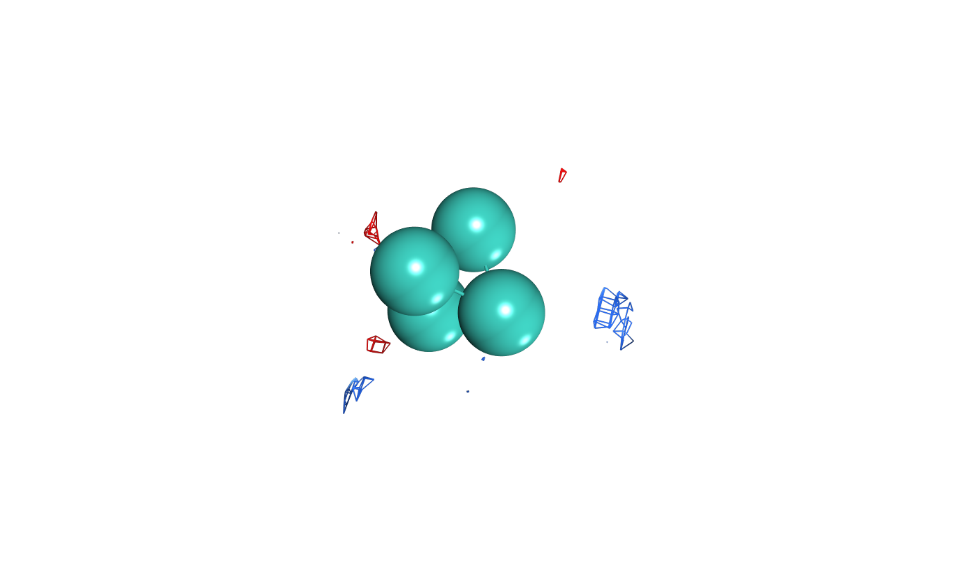  **(d)** |

**Fig. S2.** Pb nanoparticle (a) and its interaction with HAp-PEG 6000 visualized via electron density, contact and electrostatic maps. The Pb nanoparticle is depicted in the vdW spheres. The hydrogen atoms are omitted for all the maps. Electrostatically positive regions are, by default colored blue, negative regions, red and neutral regions, white

|  |  |
| --- | --- |
| (c) |  |
|  | |

**Fig. S3.** The effect of various parameters on the removal (%) of Pb^+2^ by of the two composite (a) pH value (b) foam dose (c) initial [Pb^+2^] (d) temperature and (e) time

| (a) |  |
| --- | --- |

**Fig. S4.** Langmuir (a) and Freundlich (b) adsorption plots of Pb^+2^ ions HAp and HAp-PEG 6000 at different temperatures.

| (a) |  |
| --- | --- |
| (c) | |

**Fig. S5.** Kinetic plots of Pb^+2^ adsorption by composite **a)** Pseudo first-order, **b**) Pseudo-second order, and **c)** Intraparticle diffusion model.

**Fig. S6.** Adsorption thermodynamics of Pb^+2^ ions onto HAp and HAp-PEG 6000.

**Fig. S7.** plot of Liquid film diffusion model for the adsorption of Pb^+2^ by HAp and HAp-PEG 6000


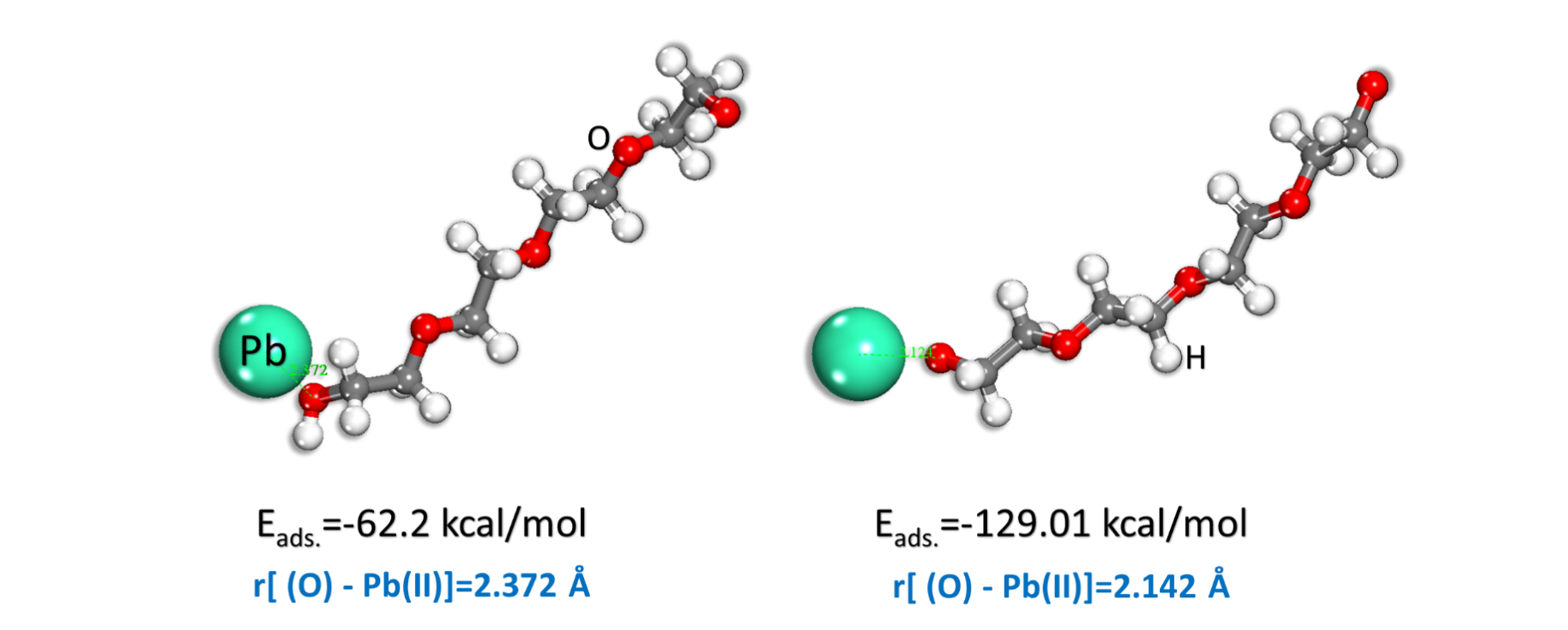


**Fig. S8**. Optimized geometries, adsorption energies and r[(O) – Pb^+2^] distances for the interaction of Pb^+2^ ions and the PEG6000 in neutral and protonated state.


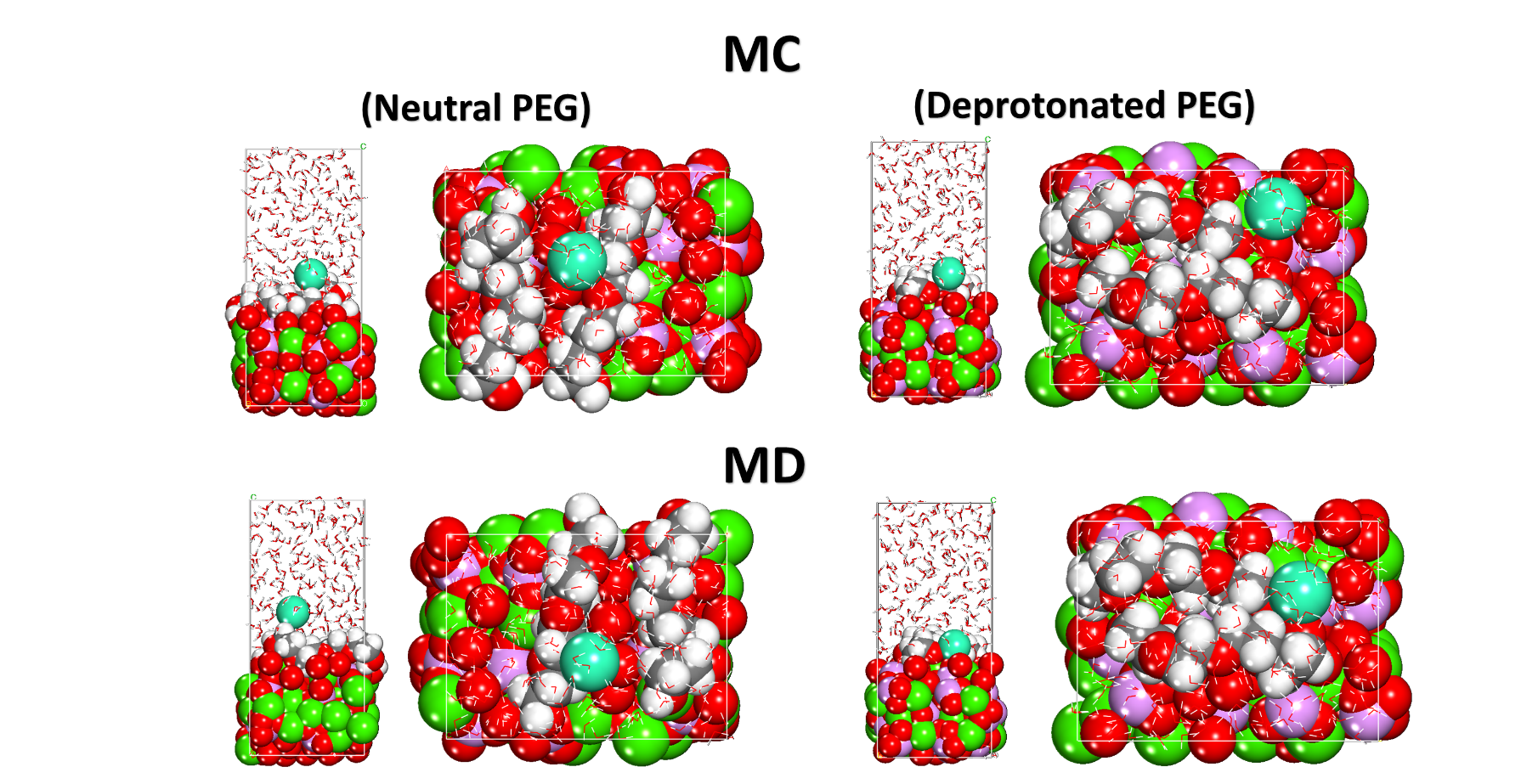


**Fig. S9**. Lowest energy geometries derived from MC and MD for the Pb^+2^ ions adsorbed onto interface of Hap /PEG6000 (in neutral and deprotonated form).


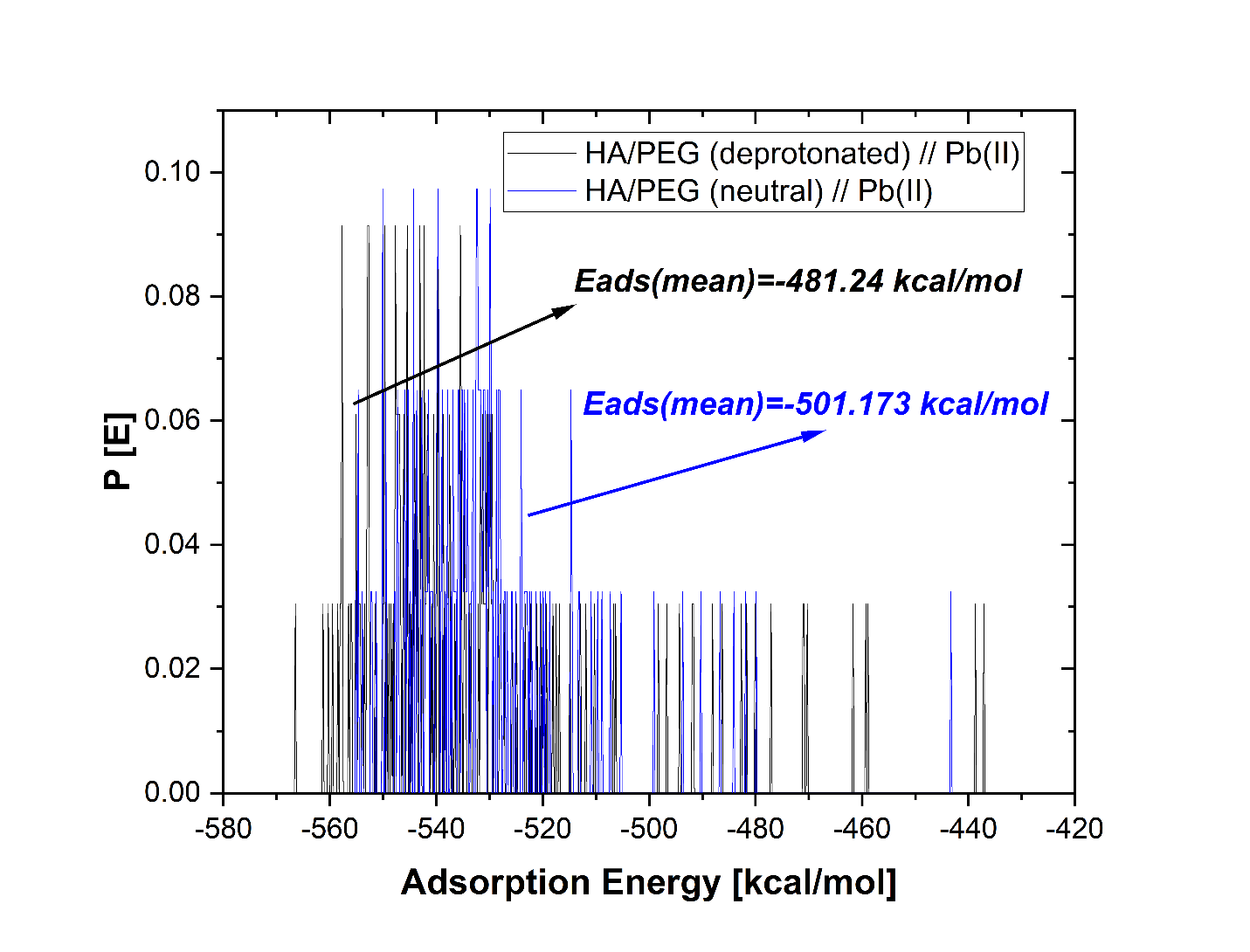


**Fig. S10.** Probability distribution of the adsorption energies from MC for the Pb^+2^ ions adsorbed onto interface of Hap

/PEG6000 (in neutral and deprotonated form).
